# Enhancing Patient Experience in Sarcoma Core Biopsies: The Role of Communication, Anxiety Management, and Pain Control

**DOI:** 10.3390/cancers16233901

**Published:** 2024-11-21

**Authors:** Ruben Jaeger, Nasian Mosku, Daniela Paganini, Georg Schelling, Kim van Oudenaarde, Anna L. Falkowski, Roman Guggenberger, Gabriela Studer, Beata Bode-Lesniewska, Philip Heesen, Bruno Fuchs

**Affiliations:** 1 Faculty of Health Sciences and Medicine, University of Lucerne, Frohburgstrasse 3, 6002 Luzern, Switzerland; 2 Sarcoma Center, Department of Orthopedics and Trauma Surgery, LUKS University Hospital Lucerne, 6000 Luzern, Switzerland; 3 Sarcoma Center, Department of Orthopedics and Traumatology, Cantonal Hospital Winterthur, 8400 Winterthur, Switzerland; 4 Department of Paediatric Surgery, University Children’s Hospital Zurich, University of Zurich, 8008 Zurich, Switzerland; 5 Medical Faculty, University of Zurich, 8032 Zurich, Switzerland; 6 Clinic for Radiology and Nuclear Medicine, Cantonal Hospital Winterthur, Brauerstrasse 15, 8401 Winterthur, Switzerland

**Keywords:** sarcoma, core biopsy, patient-reported outcomes, anxiety management, pain control, communication strategies, healthcare quality, PROMs, PREMs, invasive procedures, Invasive Procedure Assessment (IPA)

## Abstract

**Background/Objectives:** This study evaluates the effectiveness of communication strategies and pain management protocols to enhance patients’ experiences during sarcoma core biopsies. Recognizing the complexity and anxiety associated with sarcoma diagnoses, this research aims to assess the utility of the Invasive Procedure Assessment (IPA) tool, focusing on its ability to identify areas for improvement through Patient-Reported Outcome Measures (PROMs) and Patient-Reported Experience Measures (PREMs). **Methods:** Conducted at two Integrated Practice Units (IPUs) within the Swiss Sarcoma Network, this study involved 282 consecutive patients who underwent core biopsies. One week post-procedure, patients completed the IPA questionnaire, which included PROMs and PREMs. Statistical analyses explored correlations between physician communication, patient understanding, anxiety, pain, and overall satisfaction. **Results:** The IPA tool effectively captured patient perspectives on sarcoma core biopsies. A significant positive correlation (rho = 0.619, *p* < 0.0001) was found between effective physician communication and patient understanding, which was associated with reduced anxiety and pain. Higher anxiety levels strongly correlated with increased pain perception (rho = 0.653, *p* < 0.0001), emphasizing the need for anxiety management. Patients with malignant tumors reported lower overall satisfaction compared to those with benign tumors (*p* = 0.0003), highlighting the need for tailored communication and pain management strategies. The data also suggested that clear communication might mitigate overly negative subjective impressions, such as concerns about wound complications. **Conclusions:** Effective communication and anxiety management are essential for improving patient satisfaction during sarcoma core biopsies. The study emphasizes the need for personalized care strategies tailored to tumor characteristics and patient demographics. Future research should validate these findings in diverse healthcare settings and explore the broader application of the IPA tool across different cultural and demographic contexts.

## Simple Summary

This study emphasizes the crucial role of effective communication to improve patients’ understanding and satisfaction during biopsy procedures. It highlights the importance of managing patient anxiety and fear, which can directly impact pain experiences. The study also identifies areas where real-time pain management can be enhanced and underscores the need for clear and accurate patient education to address concerns after the procedure.

## 1. Introduction

The diagnosis of sarcomas poses significant challenges due to their heterogeneity and overlap of clinical features with other benign and malignant conditions [1]. Accurate diagnosis is crucial for effective treatment planning and improving patient outcomes. Core needle biopsies are essential in confirming the diagnosis of sarcoma, as they provide sufficient tissue samples for histopathological evaluation while minimizing procedural risks [2]. Studies have shown that core needle biopsies have high diagnostic accuracy and are critical for differentiating between malignant and benign lesions, ensuring that appropriate management strategies are implemented [1,3,4].

The importance of Patient-Reported Outcome Measures (PROMs) and Patient-Reported Experience Measures (PREMs) in healthcare is increasingly recognized [5]. These tools capture patients’ perspectives on their health and care, thereby informing patient-centered care and improving healthcare quality by addressing both clinical outcomes and patient experiences [5]. PROMs and PREMs help clinicians understand the impact of illness and treatment from the patient’s point of view, which is essential for tailoring treatment. Notably, the development of disease-specific instruments, such as sarcoma-specific PROMs described by Mosku et al., emphasizes the need for tools that reflect the unique challenges faced by sarcoma patients, including heterogeneity of the disease and complexity of its treatment [6]. Incorporating such tailored PROMs and PREMs into clinical practice has been shown to be associated with better clinical outcomes, improved patient satisfaction, and enhanced communication between patients and healthcare providers [5,7,8,9]. The sarcoma-specific instrument, in particular, underscores the potential of PROMs to provide comprehensive longitudinal assessments, thus facilitating shared decision-making and enhancing quality of care throughout the entire care cycle [6].

Patients undergoing biopsy procedures often face challenges such as fear, pain, and post-procedural concerns. Anxiety about the procedure is common, as patients fear the unknown and potential pain associated with the biopsy [10,11,12]. This anticipatory anxiety can amplify pain perception, a phenomenon known as the nocebo effect, where negative expectations lead to worse outcomes [13,14]. Despite the use of local anesthesia, many patients report significant pain during procedures like bone marrow and prostate biopsies [15,16]. Effective pain management strategies are crucial to minimize discomfort and improve the patient’s tolerance of the procedure [11]. Post-procedural concerns, such as complications related to bleeding, infection, and wound healing, can further increase anxiety and decrease satisfaction [17].

Thorough pre- and post-procedural education helps to mitigate these concerns by setting appropriate expectations, significantly reducing patient anxiety and overall stress [18,19,20].

Inadequate pain management and poor communication can heighten anxiety and create a negative perception of the procedure. Conversely, comprehensive information and effective pain relief are associated with higher satisfaction and better experiences. This communication is vital for improving patient’s comfort and satisfaction [11,18,21,22,23,24].

The rationale for this study is to address significant gaps in understanding and managing patient experiences during sarcoma core biopsies. While PROMs and PREMs have been widely used in cancer care, their application in sarcoma care and biopsies specifically remains under-examined. This study aims to evaluate the effectiveness of current communication strategies during sarcoma core biopsies, focusing on their impact on the reduction in patient anxiety and improving the overall perception of the procedure. Additionally, it seeks to assess the adequacy of pain management protocols in minimizing patient discomfort and enhancing satisfaction. Identifying specific areas for improvement in communication and pain management to optimize patient experiences and outcomes is also a key objective. Addressing these objectives will provide evidence-based recommendations, enhancing patient care and leading to better outcomes within the healthcare system.

## 2. Materials and Methods

This study was conducted between June 2021 and March 2024 at two Integrated Practice Units of the Swiss Sarcoma Network in Switzerland (IPU-A; IPU-B). A total of 305 consecutive participants were recruited. Inclusion criteria encompassed all patients with a suspicion of sarcoma who were undergoing an invasive core biopsy procedure under local anesthesia, with no restrictions on demographics. Due to incomplete data sets, 23 patients were excluded, leaving a final cohort of 282 participants.

Demographics, the anatomical region, and the biological behavior of the tumors are summarized in Table 1. Tumors were classified using a Comprehensive Sarcoma Classification (CSC) system that operates on two key dimensions [25]:

1. **Anatomical Regions**:
  - **Axial Regions**: Includes the head and neck, trunk, and visceral regions (further divided into intraperitoneal and retroperitoneal).
  - **Appendicular Regions**: Encompasses the upper extremities and lower extremities.
2. **Tissue of Origin**:
  - **Superficial Soft Tissue (SST)**: Tumors originating in tissues close to the skin or epifascial.
  - **Deep Soft Tissue (DST)**: Tumors originating in deeper tissues, such as muscles or below the superficial fascia.
  - **Bone**: Tumors originating from or within bone structures.

**Figure 1:**
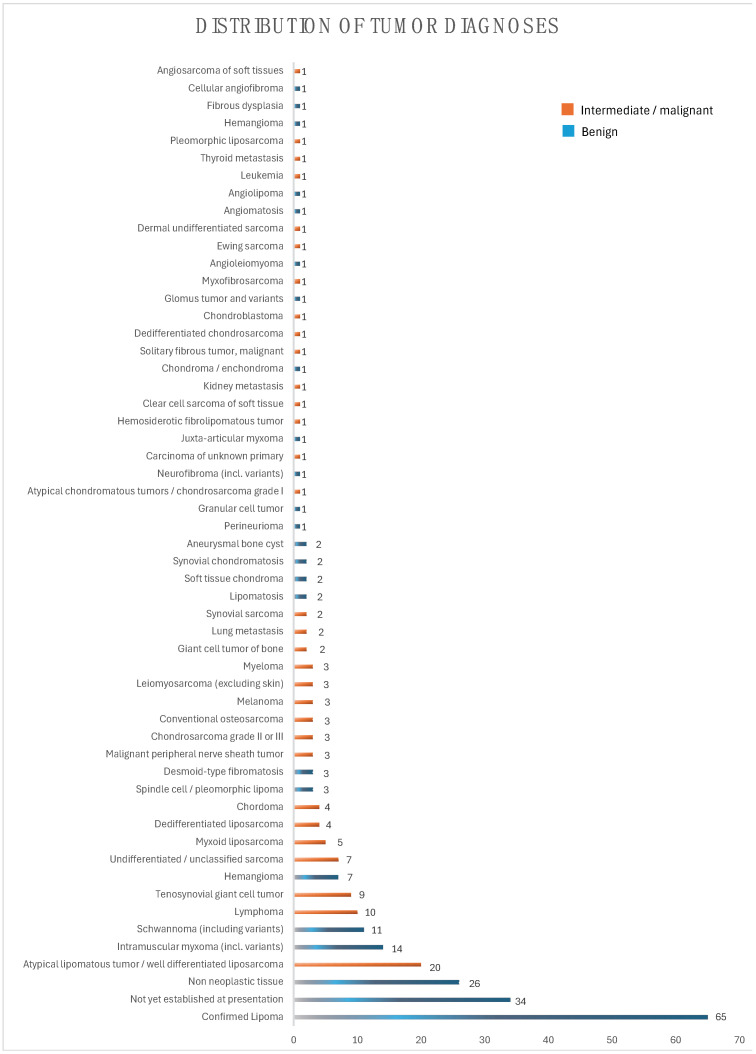
Distribution of diagnoses according to the results of biopsy at the time of presentation (n).

To provide a visual representation of the types of tumors observed in this study, Figure 1 illustrates the distribution of tumor diagnoses among the participants.

### 2.1. Creating the IPA Questionnaire for Detailed PREM and PROM Evaluation

Our Invasive Procedure Assessment (IPA) Questionnaire was developed to capture patient-reported experiences and outcomes related to invasive core biopsy procedures (Table 2). The aim was to create a comprehensive and balanced tool that would assess both positive and negative aspects of the patient’s experiences and outcomes during the biopsy process. The following four categories were evaluated: procedure perception, overall experience and comfort, pain and medication analysis, and post-procedural outcomes.

The final questionnaire consisted of 13 questions and the EQ VAS scale, which is a vertical visual analog scale ranging from 0 to 100, with endpoints labeled ‘The worst health you can imagine’ and ‘The best health you can imagine’. This scale allows for a standardized measurement of the patient’s overall health perception [26]. Additionally, Patient-Reported Experience and Outcome Measures (PREMs and PROMs) were assessed using a standardized section of the questionnaire, with questions rated on a scale from 1 to 10. This part of the questionnaire was completed by patients one week after the biopsy, immediately before their diagnosis was explained.

By carefully selecting the aspects integrated into our questionnaire, we aimed to develop a comprehensive and practical tool suitable not only for assessing biopsy procedures but also other invasive procedures in an outpatient setting. The initial application of the questionnaire at multiple IPUs within the Swiss Sarcoma Network serves as a first step in a formal validation process [27], ensuring that it effectively captures relevant data to improve patient care and communication strategies.

Questions composing the questionnaire:
Q1: Did you understand the reason and necessity for the procedure?
1 (not at all understood)—10 (fully understood)
Q2: Was the procedure well explained by the physician who performed it?
1 (not at all explained)—10 (very well explained)
Q3: How well did you understand what was done during the procedure?
1 (not at all understood)—10 (perfectly understood)
Q4: How strong was your fear from the procedure?
1 (strongest fear ever experienced)—10 (no fear)
Q5: Consider the entire procedure, did you feel comfortable with the team and the room set-up?
1 (not at all comfortable)—10 (very comfortable)
Q6: How would you rate the entire organization process of the procedure?
1 (not at all good)—10 (very good)
Q7: How badly did you feel the pain during the procedure?
1 (strongest pain ever experienced)—10 (no pain)
Q8: Did the pain experienced during the procedure match your expectation?
1 (not at all matched)—10 (perfectly matched)
Q9: How satisfied were you with the medication you received during the procedure?
1 (not at all satisfied)—10 (very satisfied)
Q10: Was the pain well controlled during the first 24–48 h after the procedure?
1 (not at all controlled)—10 (very well controlled)
Q11: Was the observation period after the procedure long enough until dismissal?
1 (it was not long enough, I felt stressed)—10 (it was long enough, I did not feel stressed at all)
Q12: Was there any bleeding after the procedure?
1 (heavy bleeding)—10 (no bleeding)
Q13: Did you experience other local problems after the procedure (hematoma, redness, infection, swelling, other)?
1 (many other problems)—10 (no problems)

### 2.2. Data Analyses

The variable “overall satisfaction” (OS) was defined as the mean of all scores from the respective IPA questions:

$$OS=\frac{Q1+Q2+Q3+Q4+Q5+Q6+Q7+Q8+Q9+Q10+Q11+Q12+Q13}{13}$$

Data were analyzed using the R statistical software version R 4.0.2. Descriptive statistics were computed for all variables, including means and standard deviations (SDs) for normally distributed data, and medians with interquartile ranges (IQRs) for non-normally distributed data. Normality was assessed through a visual inspection of histograms and the Shapiro–Wilk W test. For normally distributed data, parametric tests were applied, while non-normally distributed data were analyzed using non-parametric methods. Variables significantly deviating from normality were transformed to correct skewness before further analysis. Differences in PROMs by gender, anatomic region, and tumor localization were examined using the Wilcoxon rank-sum test or Kruskal–Wallis test, depending on the number of groups compared. In cases where the Kruskal–Wallis test indicated significant differences, post hoc pairwise comparisons were conducted using the Dunn test with Bonferroni correction to control for multiple testing. Spearman’s rank correlation was also computed. Significance was set at *p* < 0.05.

## 3. Results

### 3.1. Differences Depending on Gender, Anatomic Region of the Tumor, Origin of Lesion, and Biological Behavior of the Tumor

Analysis by gender, different anatomic regions, or tissue of origin revealed no statistically significant differences in most PROMs, including necessity (Q1), explanation (Q2), understanding (Q3), fear (Q4), team room (Q5), organization (Q6), pain perception (Q7), pain expectation (Q8), medication (Q9), pain control (Q10), observation (Q11), bleeding (Q11), wound complication (Q13), EQ VAS, and overall satisfaction. However, a notable exception was found in the PROM “understanding” (Q3), where patients with retroperitoneal tumors reported significantly lower scores compared to those with tumors in the head and neck (*p* = 0.032), lower extremity (*p* = 0.024), and trunk regions (*p* = 0.013). This may reflect the added complexity or less visible nature of these tumors.

Furthermore, the PROM “fear” (Q4) also showed significant variation by anatomic region (*p* = 0.015), with patients having trunk tumors reporting higher fear (lower scores in Q4) compared to those with upper extremity tumors (*p* = 0.006). This increased fear in patients with trunk tumors may be associated with the proximity of vital organs in these cases.

Statistically significant differences were observed in the PROM “observation” (Q11) depending on the anatomic region of the tumor (*p* = 0.023). Specifically, patients with tumors in the trunk reported higher observation time scores compared to those with tumors in the upper extremity (*p* = 0.009), which may reflect greater caution or concern in clinical observation for these tumors.

Additionally, a significant difference was noted in the EQ VAS score depending on the biology of the tumor, with patients diagnosed with malignant tumors reporting lower overall health scores compared to those with benign tumors (*p* = 0.0003). This underscores the profound impact of malignant pathology on patients’ health status and perception, highlighting the potential need for additional supportive care measures for these patients.

### 3.2. Overall Analyses of IPA PROMs and PREMs

**Figure 2:**
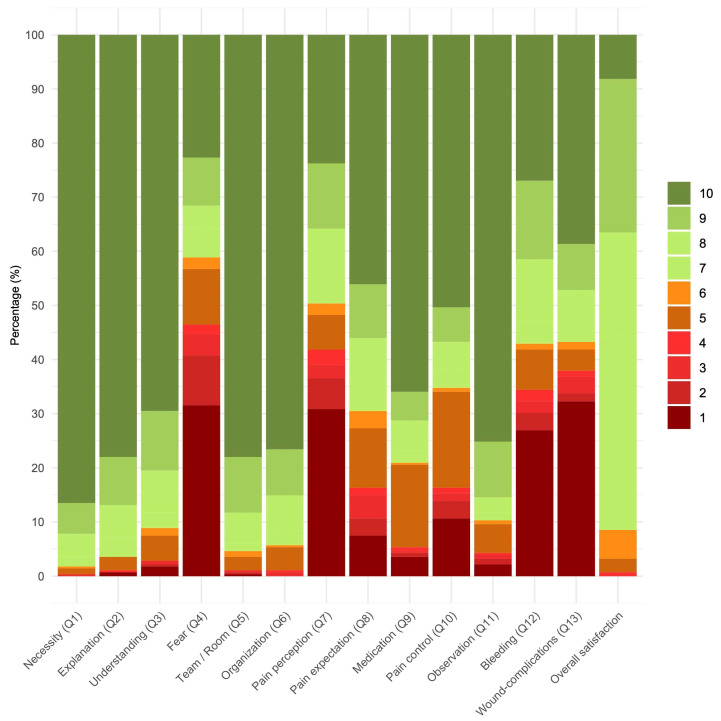
Patient reported outcome/experience measures of all participants (n = 282).

Table 3 provides a detailed overview of the Patient-Reported Outcome Measures (PROMs) and Patient-Reported Experience Measures (PREMs) for all participants (n = 282), showing the total number of participants who reported each score and the corresponding percentage. This table allows for a granular understanding of the distribution of responses across different domains, including pain, satisfaction with medication, observation period, bleeding, local problems, pain control, understanding of the biopsy necessity, fear, explanation by the physician, understanding of the procedure, comfort with the team and room setup, and the overall organization process. See Figure 2 for a visual representation of these measures across all participants.

### 3.3. Results According to IPA Categories

#### 3.3.1. Procedure Perception

The results indicated generally positive perceptions regarding the procedure, with high scores for understanding the necessity (median score 10, IQR 10–10), explanation (median score 10, IQR 10–10), and procedural understanding (median score 10, IQR 9–10); a significant level of fear was reported, with 31.6% of patients expressing the highest level of fear (score 1 on a scale of 1–10). This fear could stem from concerns about the procedure itself or the potential consequences, such as being diagnosed with a malignant lesion, as indicated by the distribution of lower scores in the “fear” domain (median score 5, IQR 1–9). The distinction between these sources of fear is essential for developing targeted strategies to alleviate anxiety.

#### 3.3.2. Overall Experience and Comfort

The overall experience and comfort of patients were rated highly, with the median scores for satisfaction with the team and room setup at 10 (IQR 10–10) and 78% of patients rating their experience as the highest possible score (score 10) (Table 4, Q5). The organization of the procedure also received positive feedback, with a median score of 10 (IQR 10–10), and 76.6% of patients rated the organization as excellent (score 10) (Table 3, Q6). These high ratings indicate that the environment and professionalism of the medical staff greatly contributed to patient comfort. There were no immediate areas identified for improvement in this category, suggesting that the current practices are effective in ensuring a supportive procedural environment.

#### 3.3.3. Pain and Medication Analysis

Pain management emerged as a crucial area with room for improvement. Patients reported experiencing high levels of pain during the procedure, with a median score of 6 (IQR 1–9), where 30.8% of patients rated their pain at the highest level (score 1) (Table 3, Q7). This pain often matched their expectations, with a median score of 9 (IQR 5–10) for pain expectation alignment (Table 3, Q8), suggesting a potential “self-fulfilling prophecy” effect. While medication was generally considered sufficient, with a median score of 10 (IQR 8–10) and 66% of patients rating their satisfaction with medication at the highest level (score 10) (Table 3, Q9), pain control during the procedure itself showed room for improvement. Pain control post-procedure was relatively well-rated, with a median score of 10 (IQR 5–10), and 50.4% of patients felt that their pain was very well controlled within the first 24–48 h after the procedure (Table 3, Q10). These data indicate that although post-procedural pain control was effective for most patients, enhancing intra-procedural pain management could further improve patient experiences.

#### 3.3.4. Post-Procedural Outcomes

Post-procedural outcomes were largely positive, with high satisfaction regarding the observation period after the procedure, as indicated by a median score of 10 (IQR 10–10) and 75.2% of patients rating the observation period at the highest level (score 10) (Table 3, Q11). Despite this, 27% of patients expressed concerns about post-procedural bleeding, with scores reflecting the presence of bleeding rated at a median of 8 (IQR 1–10) (Table 3, Q12). Additionally, concerns about wound complications were noted, with a median score of 8 (IQR 1–10) and 32.3% of patients reporting high levels of worry about wound-related issues (score 1) (Table 3, Q13). Importantly, no cases required surgical revisions or therapeutic interventions, suggesting that these concerns were likely due to misaligned expectations. Enhancing patient education on what to expect post-procedure could help to mitigate these concerns and further improve overall satisfaction.

### 3.4. Correlations Between PROMs

#### 3.4.1. Correlation Between Physician Explanation and Various PROMs

A significant positive correlation was observed between the PROM related to the physician’s explanation and the PROM related to understanding the procedure (rho = 0.619, *p* < 0.000), indicating that clear and comprehensive explanations from physicians strongly enhance patient comprehension, which is crucial for reducing anxiety and improving overall patient experience. Additionally, a significant positive correlation was found between the explanation and the understanding of the necessity of the biopsy (rho = 0.288, *p* < 0.000), further emphasizing the importance of clear communication in ensuring that patients understand the rationale behind the procedure.

While the correlation between physician’s explanation and fear of biopsy was negative, it did not reach statistical significance (rho = −0.117, *p* = 0.052), suggesting that although better explanations may help reduce fear, this effect might be limited or require more targeted interventions to achieve significant anxiety reduction. Furthermore, significant positive correlations were observed between the explanation provided by the physician and satisfaction with the setup (rho = 0.573, *p* < 0.000), satisfaction with the organization (rho = 0.389, *p* < 0.000), and overall satisfaction (rho = 0.241, *p* < 0.000), highlighting that effective communication not only improves understanding but also enhances overall procedural satisfaction and the perception of care quality.

#### 3.4.2. Correlations Between Fear of the Biopsy and Pain

There was a significant positive correlation between fear of the biopsy and experienced pain during the biopsy (rho = 0.653, *p* < 0.001). Additionally, there was a significant negative correlation between fear of biopsy and pain control after biopsy (rho = −0.287, *p* < 0.001) (Table 5).

#### 3.4.3. Correlations Between Expected Pain, Experienced Pain, and Pain Control

There was no significant correlation between the expected pain and the experienced pain during the biopsy. However, there was a significant weak negative correlation between experienced pain during biopsy and pain control for 24 h post-procedure (rho = −0.257, *p* < 0.001) (Table 5).

### 3.5. Comparison of Patient-Reported Outcome Measures Between IPU-A and IPU-B

Upon comparing the two Integrated Practice Units (IPUs), IPU-A and IPU-B, there were no significant demographic differences found between patient populations, including age, gender, and sarcoma classification. Despite statistically significant differences in several areas, the actual mean and median values between IPU-A and IPU-B were quite similar, indicating that these differences may have limited practical impact (Table 5). For instance, patients at IPU-B reported lower levels of fear and pain during the biopsy, as well as higher overall satisfaction, compared to those at IPU-A. On the other hand, IPU-A scored slightly better in the procedural setup and patients’ overall health perception as measured by the EQ VAS scale. These findings indicate that both institutions provide a high standard of care, with only minor differences in specific aspects of patient experience (Table 4; Supplementary Figures S1 and S2).

## 4. Discussion

This study evaluated the use of Patient-Reported Outcome Measures (PROMs) and Patient-Reported Experience Measures (PREMs) in assessing sarcoma biopsy procedures, with a focus on identifying areas for improvement [5,8,9,28,29,30]. Given the unique challenges in sarcoma care, the development and application of sarcoma-specific instruments, as highlighted by Mosku et al., are crucial for accurately capturing patient experiences and outcomes, especially when dealing with such a heterogeneous and complex disease [4,6]. Data from 282 patients undergoing core biopsies with the suspicion of sarcoma at two Integrated Practice Units (IPUs) of the Swiss Sarcoma Network (SSN) highlighted generally high patient satisfaction, but also identified critical areas needing enhancement, particularly in managing anxiety, pain, and post-procedural concerns such as bleeding or wound complications.

Our findings demonstrate a significant positive correlation between detailed explanations provided by physicians and patients’ understanding of the procedure and the necessity of the biopsy. This correlation highlights the critical role of effective communication in reducing anxiety and pain during the procedure, suggesting that clear and compassionate communication may directly influence patients’ physiological responses, potentially through mechanisms such as the modulation of stress and pain pathways. This is consistent with the broader literature on the impact of communication in medical settings [10,11,18,19,31]. These findings underscore the importance of clear and compassionate communication in improving patient outcomes, as has been observed in studies on breast and lung biopsies [10,11,31]. It appears that this positive effect of communication extends to the sarcoma patient population as well.

Despite these positive correlations, this study also revealed areas where patient experiences could be improved, particularly in managing intra-procedural pain. The significant correlation between fear and pain perception suggests that anxiety management is not only important for emotional well-being but may also have a direct impact on physical pain experiences during the procedure. While post-procedural pain management was generally effective, controlling pain during the biopsy remains a significant challenge. This aligns with previous research on the persistent pain experienced during procedures such as bone marrow and prostate biopsies, despite the use of local anesthesia [15,16,32]. The “self-fulfilling prophecy” nocebo effect, where patients’ expectations of pain may lead to heightened pain perception, further complicates this issue [14]. This relationship underscores the importance of addressing psychological factors, such as fear and anxiety, as part of a comprehensive pain management strategy. Implementing advanced pain relief methods, such as music therapy or virtual reality, particularly for patients experiencing high levels of anxiety, may be considered to improve patient comfort during these procedures [33,34].

The comparison between the two IPUs, while revealing some statistically significant differences in fear, pain, and overall satisfaction, suggest that these differences are nuanced rather than indicative of major disparities in care quality. This finding is intriguing, especially given the similar demographic and cultural contexts of the two sites. Future research could explore whether these subtle differences might be more pronounced in more culturally or demographically diverse settings, which could provide valuable insights into how various factors influence Patient-Reported Outcome Measures.

Additionally, this study found that fear of the biopsy was a significant predictor of pain perception, a relationship well documented in clinical settings involving both acute and chronic pain [35,36,37,38,39]. This finding suggests that fear may amplify the sensory experience of pain, possibly through heightened activation of the central nervous system’s pain pathways, and indicates that psychological factors can significantly influence the physical experience of pain. This correlation highlights the need for targeted interventions to manage patient anxiety before and during the procedure. Strategies that incorporate cognitive-behavioral approaches, mindfulness, or other anxiety reduction techniques could be effective in mitigating this fear–pain relationship. Improving patient education about post-procedural outcomes, particularly regarding common but minor complications like hematomas, could also enhance overall satisfaction. Many patients expressed concern about bleeding and wound complications, likely due to misaligned expectations. Educating patients on what to expect after the procedure could alleviate these concerns and reduce anxiety [10,17,18].

While the sample size of this prospective study was adequate for the purposes of the analysis, larger studies could provide more robust data and help confirm the findings. However, the reliance on self-reported data introduces potential biases, such as recall bias or social desirability bias, which may affect the accuracy and reliability of the findings. Moreover, this study’s focus on a specific demographic group in Switzerland limits the generalizability of the results to other populations or healthcare systems. The healthcare context in Switzerland, characterized by specific organizational and cultural factors, may not be directly comparable to other settings, which could limit the applicability of the findings. Future research should include more diverse populations and healthcare settings to explore how these findings translate across different contexts. Additionally, addressing potential selection bias by ensuring a more representative sample of patients, including those from varied socioeconomic and cultural backgrounds, would enhance the validity of future studies [40,41].

To fully understand the potential of the Invasive Procedure Assessment (IPA) tool, future studies should explore its application in a variety of settings and contexts. Testing the IPA across different healthcare environments—such as diverse geographic locations, cultural backgrounds, and medical specialties—would help validate its effectiveness and reveal its versatility. This broader application could uncover specific insights about patient experiences in different scenarios, demonstrating how the IPA can be an effective tool for assessing and improving medical procedures in various settings. This approach would highlight the IPA’s capacity to enhance patient-centered care on a larger scale.

## 5. Conclusions

This study underscores the critical importance of effective communication and personalized pain management in enhancing patient experience during sarcoma core biopsies. Integrating PROMs and PREMs into routine practice facilitates a deeper understanding of patient needs and highlights areas for improvement. By addressing both psychological and physical aspects of care, we can significantly improve patient-centered outcomes. Future research should validate these findings in diverse settings and explore the broader application of the IPA tool to further optimize patient care.

## Figures and Tables

**Table 1:** Age, gender, IPU, and sarcoma classification and biological behavior of the tumor among all participants (n = 282).

Parameter	Values	
Age (years)	55.6 ± 17.6 *	
Male/Female (n (%))	147 (52)/135 (48)	
Integrated Practice Units (IPUs) (n (%)) ^1^		
IPU-A	146 (52)	
IPU-B	136 (48)	
Anatomic region (n (%)) ^2^		
Trunk	85 (30)	
Head/Neck	7 (2)	
Visceral retroperitoneal	7 (2)	
Visceral intraperitoneal	3 (1)	
Upper extremity	39 (14)	
Lower extremity	141 (50)	
Origin of lesion (n (%))		
DST ^3^	192 (68)	
SST ^4^	48 (17)	
Bone	43 (15)	
Benign/malignant (n (%)) ^5^	181 (64)/101 (36)	

* Mean ± SD. ^1^ Defines in which IPU the study was conducted. ^2^ Anatomic region. ^3^ Deep soft tissue. ^4^ Superficial soft tissue. ^5^ Defines the biological behaviors of the tumors.

**Table 2:** Categorization of PROMs and PREMs in the IPA questionnaire.

PREMs		PROMs	
Procedure Perception	Overall Experience and Comfort	*Pain and Medication Analysis*	Post-Procedural Outcomes
**Necessity (Q1)** **Explanation (Q2)** **Understanding (Q3)** **Fear (Q4)**	**Team/Room (Q5)** **Organization (Q6)**	**Pain perception (Q7)** **Pain expectation (Q8)** **Medication (Q9)** **Pain Control (Q10)**	**Observation (Q11)** **Bleeding (Q12)** **Wound complications (Q13)**

**Table 3:** Summary of PROMs and PREMs for all participants (n = 282), showing scores and percentages.

PROM Domain	Median (IQR)	0 (n(%))	1 (n(%))	2 (n(%))	3 (n(%))	4 (n(%))	5 (n(%))	6 (n(%))	7 (n(%))	8 (n(%))	9 (n(%))	10 (n(%))
Q1 necessity	10 (10–10)	0 (0)	0 (0)	0 (0)	1 (0.4)	0 (0)	3 (1.1)	1 (0.4)	5 (1.8)	12 (4.3)	16 (5.7)	244 (86.5)
Q2 explanation	10 (10–10)	0 (0)	2 (0.7)	0 (0)	0 (0)	1 (0.4)	7 (2.5)	0 (0)	10 (3.5)	17 (6.0)	25 (8.9)	220 (78.0)
Q3 understanding	10 (9–10)	0 (0)	5 (1.8)	1 (0.4)	2 (0.7)	0 (0)	13 (4.6)	4 (1.4)	8 (2.8)	22 (7.8)	31 (11.0)	196 (69.5)
Q4 fear	5 (1–9)	0 (0)	89 (31.6)	26 (9.2)	11 (3.9)	5 (1.8)	29 (10.3)	6 (2.1)	15 (5.3)	12 (4.3)	25 (8.9)	64 (22.7)
Q5 team/room	10 (10–10)	0 (0)	1 (0.4)	1 (0.4)	1 (0.4)	0 (0)	7 (2.5)	3 (1.1)	4 (1.4)	16 (5.7)	29 (10.3)	220 (78.0)
Q6 organization	10 (10–10)	0 (0)	0 (0)	0 (0)	2 (0.7)	1 (0.4)	12 (4.3)	1 (0.4)	8 (2.8)	18 (6.4)	24 (8.5)	216 (76.6)
Q7 pain perception	6 (1–9)	0 (0)	87 (30.8)	16 (5.7)	7 (2.5)	8 (2.8)	18 (6.4)	6 (2.1)	12 (4.3)	27 (9.6)	34 (12.1)	67 (23.8)
Q8 pain expectation	9 (5–10)	0 (0)	21 (7.4)	9 (3.2)	12 (4.3)	4 (1.4)	31 (11.0)	9 (3.2)	14 (5.0)	24 (8.5)	28 (9.9)	130 (46.1)
Q9 medication	10 (8–10)	0 (0)	10 (3.5)	2 (0.7)	1 (0.4)	2 (0.7)	43 (15.2)	1 (0.4)	6 (2.1)	16 (5.7)	15 (5.3)	186 (66.0)
Q10 pain control	10 (5–10)	0 (0)	30 (10.6)	9 (3.2)	4 (1.4)	3 (1.1)	50 (17.7)	2 (0.7)	10 (3.5)	14 (5.0)	18 (6.4)	142 (50.4)
Q11 observation	10 (10–10)	0 (0)	6 (2.1)	3 (1.1)	2 (0.7)	1 (0.4)	15 (5.3)	2 (0.7)	4 (1.4)	8 (2.8)	29 (10.3)	212 (75.2)
Q12 Bleeding	8 (1–10)	0 (0)	76 (27.0)	9 (3.2)	6 (2.1)	6 (2.1)	21 (7.4)	3 (1.1)	12 (4.3)	32 (11.3)	41 (14.5)	76 (27.0)
Q13 wound complications	8 (1–10)	0 (0)	91 (32.3)	4 (1.4)	9 (3.2)	3 (1.1)	11 (3.9)	4 (1.4)	4 (1.4)	23 (8.2)	24 (8.5)	109 (38.7)
Q14 overall satisfaction	8 (7–9)	0 (0)	0 (0)	0 (0)	0 (0)	2 (0.7)	7 (2.5)	16 (5.3)	100 (35.5)	54 (19.1)	80 (28.4)	23 (8.2)

**Table 4:** Analysis of PROM/PREM differences between IPU-A (n = 146) and IPU-B (n = 136).

IPA Domain	IPU-A	IPU-B	*p*-Value
Q4 fear	4.5 ± 3.7 * 3 (1–8.8) **	5.8 ± 3.7 6 (2–10)	0.002
Q7 pain perception	4.8 ± 3.7 4 (1–9)	6.4 ± 3.7 8 (1–10)	<0.001
Q5 team/room	9.4 ± 1.3 10 (9–10)	9.6 ± 1.3 10 (10–10)	0.047
Q9 overall satisfaction	7.7 ± 1.2 7 (7–9)	8.1 ± 1.3 8 (7–9)	0.003
* mean ± SD, all such values ** median (IQR), all such values			

**Table 5:** Positive and negative correlations between PROMs and PREMs. This table presents the significant positive and negative correlations between various Patient-Reported Outcome Measures (PROMs) and Patient-Reported Experience Measures (PREMs).

Positive Correlations		Negative Correlations	
PREMs/PROMs	rho-/*p*-Value	PREMs/PROMs	rho-/*p*-Value
explanation and understanding	0.619/<0.0001	explanation and fear	−0.117/0.052
explanation and necessity	0.288/<0.0001	fear and pain control	−0.287/<0.0001
fear and pain	0.653/<0.0001	pain perception and control	−0.257/<0.0001

## Data Availability

The data presented in this study are available on request from the corresponding author.

## Supplementary Materials

The following supporting information can be downloaded at: https://www.mdpi.com/article/10.3390/cancers16233901/s1, Figure S1. Patient reported outcome/experience measures at IPU-A (n = 147). Figure S2. Patient reported outcome/experience measures at IPU-B (n = 136).
