# Supplementary material for: Enhancing Patient Experience in Sarcoma Core Biopsies: The Role of Communication, Anxiety Management, and Pain Control
Source: Cancers (Basel). 2024 Nov 21;16(23):3901. doi: 10.3390/cancers16233901 (PMC11640716; doi:10.3390/cancers16233901)
Supplement: Supplementary file 1 [file cancers-16-03901-s001.zip › cancers-3206648-supplementary.pdf]

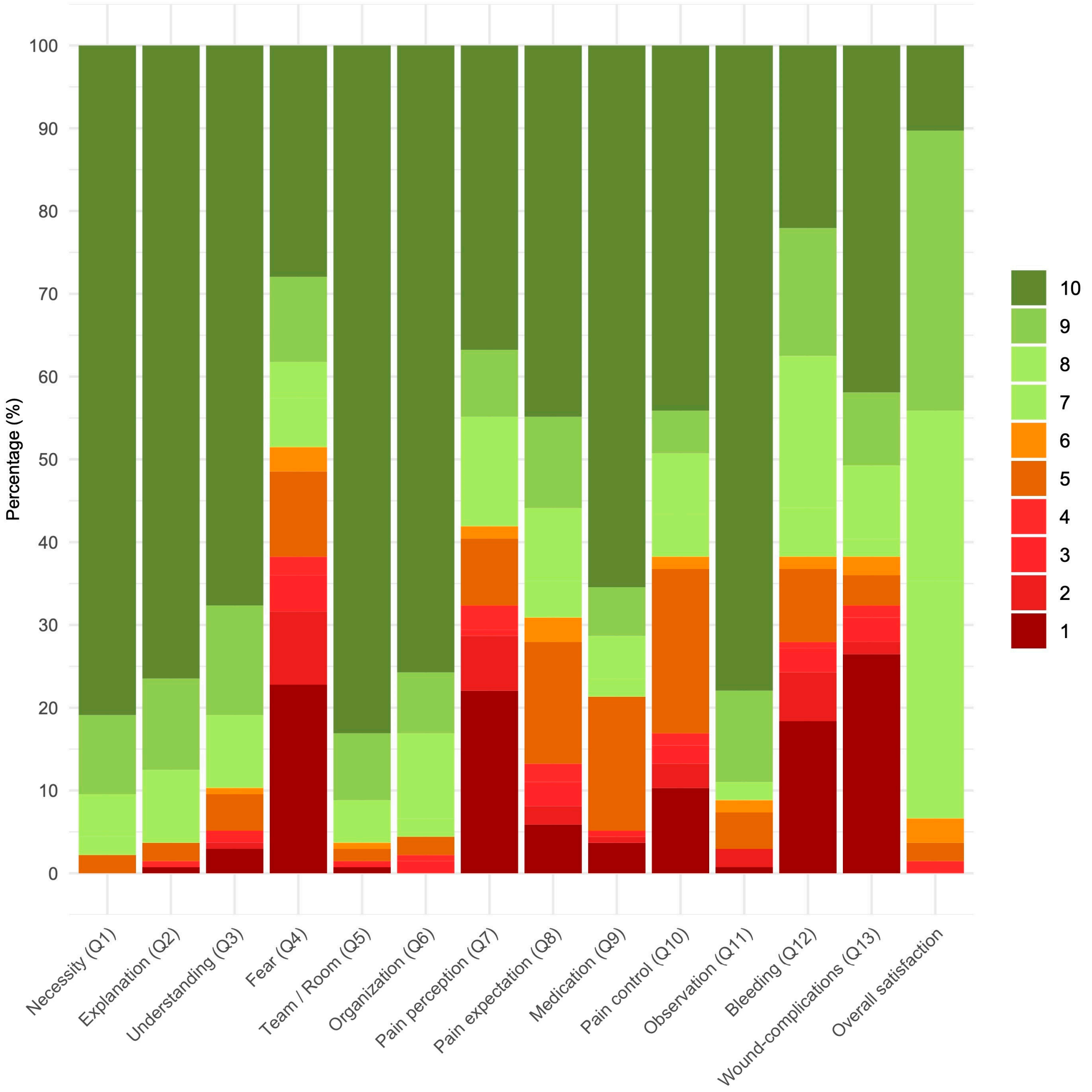

Figure S1. Patient reported outcome / experience measures at IPU-A (n = 147).

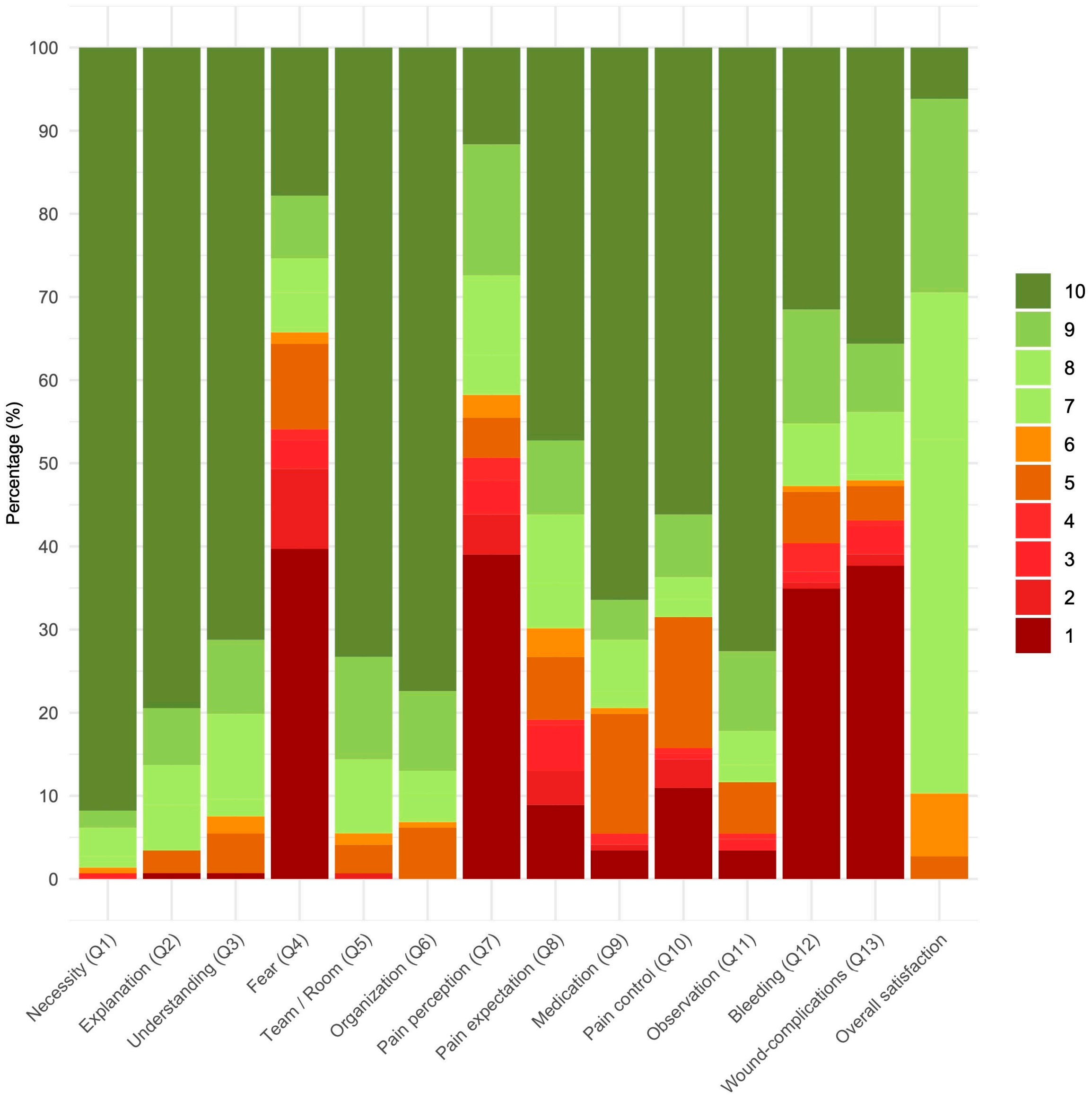

Figure S2. Patient reported outcome / experience measures at IPU-B (n = 136).
